# Endothelial dysfunction and arterial stiffness in pre-eclampsia demonstrated by the EndoPAT method

**DOI:** 10.5830/CVJA-2016-047

**Published:** 2017

**Authors:** A Meeme, M Mammen, A Namugowa, GAB Buga

**Affiliations:** Department of Human Biology, Walter Sisulu University, Mthatha, South Africa; Department of Human Biology, Walter Sisulu University, Mthatha, South Africa; Department of Human Biology, Walter Sisulu University, Mthatha, South Africa; Department of Obstetrics and Gynaecology, Walter Sisulu University, Mthatha, South Africa

**Keywords:** EndoPAT, reactive hyperaemia index, arterial stiffness, augmentation index, pre-eclempsia

## Abstract

**Objectives:**

The EndoPAT method has been used as a noninvasive method for assessing endothelial function in several non-pregnant populations. We investigated its possible use in assessing endothelial dysfunction in pre-eclampsia.

**Methods:**

Two hundred and fifteen participants were recruited and grouped as pre-eclamptic cases (105) and normotensive controls (110). Endothelial function and arterial stiffness were measured as reactive hyperaemia index and augmentation index, respectively, using the EndoPAT 2000 machine.

**Results:**

The reactive hyperaemia index was significantly lower in the pre-eclamptic group compared to the normotensive group (p < 0.05). Augmentation index on the other hand was significantly higher in the pre-eclamptic group compared to the normotensive group (p < 0.0001).

**Conclusion:**

The EndoPAT method demonstrates endothelial dysfunction and arterial stiffness in pre-eclampsia.

## Objectives

Pre-eclampsia is a pregnancy-specific multisystem disorder characterised by new-onset hypertension and proteinuria from 20 weeks’ gestation in a previously normotensive pregnant woman. It is one of the major causes of maternal and perinatal morbidity and mortality worldwide and more so in developing countries.^1^,^2^

In the complex and intriguing pathogenesis and pathophysiology of pre-eclampsia, endothelial dysfunction remains the most agreed-upon central mechanism involved, leading to clinical manifestations of the syndrome.^3^ Endothelial function in pregnancy has been assessed using several methods in different studies, including direct, indirect, invasive and non-invasive techniques.^4^

The most commonly used (gold standard) non-invasive technique in assessing endothelial function in pregnancy is the ultrasonography method called flow-mediated dilatation (FMD) of the brachial artery. The technique measures endothelial function by inducing reactive hyperaemia (which is based on nitric oxide production and bioavailability) by temporary occlusion and measuring the resultant relative increase in blood vessel diameter.^5^-^10^ This method however is user dependent, expensive and requires specialised, trained personnel to execute, and it is not readily available for routine investigational use. This means there is a need for a technique that is non-invasive, accurate, affordable and reliable to use either alone or in combination with other markers in screening patients for risk for pre-eclampsia.

The EndoPAT method is non-invasive, easy to use, userindependent and immediate, and provides automatically calculated results for assessing endothelial function.^11^ It has been used rather extensively in recent years for assessing endothelial dysfunction in non-pregnant subjects.^12^-^15^ However, its use in pregnancy and pre-eclampsia is limited to only a few studies done in Scotland and Israel.^16^,^17^ No study has reported on its use in assessing endothelial function in a rural African population.

This study set out to assess pulse-amplitude tonometry (PAT) using EndoPAT 2000 in normotensive and hypertensive pregnant women in rural Africa to determine whether it could demonstrate endothelial dysfunction associated with pre-eclampsia.

## Methods

The study was done with approval from the Bio-Ethics Committee of the Faculty of Health Sciences, Walter Sisulu University (bioethics clearance certificate No.045/010). It was a prospective case–control study conducted in Mthatha Hospital Complex, Eastern Cape, South Africa, between 2010 and 2013.

A total of 215 participants with known HIV status were recruited into the study; 105 women had pre-eclampsia (cases) and 110 were normotensive pregnant women (controls). Selection of cases was based on persistent blood pressure of ≥ 140/90 mmHg on two occasions, at least four to six hours apart or a single reading of ≥ 160/110 mmHg, and proteinuria of ≥ 1+ on at least two random specimens collected at least four hours apart (or a 24-hour urine protein of ≥ 300 mg/l) from 20 weeks of gestation, as defined by the International Society for the Study of Hypertension in Pregnancy (ISSHP). Controls were age-matched (within two to three years) and gestational age-matched (within two weeks) normotensive pregnant women attending the antenatal clinic or admitted for other obstetric conditions other than hypertension or diabetes.

Women with severe hypertension (blood pressure of ≥ 160/110 mmHg) not responding to treatment, imminent eclampsia, foetal distress, the HELLP syndrome or eclampsia and other complications requiring immediate intervention were excluded from the study. Women with known history of chronic hypertension or diabetes mellitus were also excluded from the study.

Endothelial function was assessed using the EndoPAT 2000 technique, which measures PAT using the reactive hyperaemia index (RHI, arbitrary units). Briefly, after 20 minutes’ rest in a chair inclined at an angle of about 45° at room temperature, a blood pressure cuff was placed on the non-dominant upper arm (study arm), while the other arm served as the control. The hands were placed on armchair supports with the palm side down, such that the fingers hung freely.

The EndoPAT probes were then placed on the tip of each index finger of both hands. The probes were prevented from touching any other finger or object, and were then electronically inflated. The PAT signal was continuously recorded on a personal computer during the test. Baseline pulse amplitude was measured from each fingertip for five minutes. After baseline recording of five minutes on each arm, arterial flow was then interrupted in the experimental arm by rapidly inflating the cuff to occlusion pressure of 200 mmHg or 60 mmHg plus systolic blood pressure (whichever was higher). After exactly five minutes’ occlusion, the cuff pressure was rapidly deflated, and post-occlusion recording continued for another five minutes in the experimental arm as well as the control arm.

Pulse amplitude response to hyperaemia was automatically calculated from the hyperaemia in the finger of the experimental arm as a ratio of post-deflation average pulse amplitude to the baseline average pulse amplitude (i.e. A_h_/A_h_, with A representing pulse amplitude, *h* denoting hyperaemic finger). This result was divided by the corresponding ratio from the contralateral, control hand (i.e. A_c_/A_c_, with *c* denoting the control finger) to obtain the RH–PAT ratio or PAT ratio.

The EndoPAT 2000 not only measured endothelial function with the RHI but also assessed arterial stiffness by measuring the peripheral augmentation index (PAIx) from the radial pulsewave analysis. PAIx was automatically calculated as the ratio of the difference between the early and late systolic peaks of the waveform relative to the early peak (P_2_–P_1_/P_1_), expressed as a percentage.

## Statistical analysis

Graphpad Prism 5 (GraphPad software Inc, San Diego, California) software was used for data analysis. Normality of the data distribution was evaluated by the Shapiro–Wilk and Kolmogorov and Smirnov normality tests. Data are summarised as means ± standard error of the mean (SEM) for normally distributed data and medians (interquartile range, IQR) for non-normally distributed data. The two-sample Student’s t-test was used to compare means, while the Mann–Whitney U-test was used to compare medians. Spearman’s correlation and multiple regression analyses were used to determine relationships between RHI, PAlx, baseline pulse-wave amplitude (BPWA) and maternal blood pressure.

Secondary analysis was carried out based on whether the cases had early- or late-onset pre-eclampsia and whether cases and controls were HIV positive or negative. Kruskal–Wallis and one-way ANOVA were used to compare means between the cases and controls. Statistical significance was set at p < 0.05.

## Results

The general characteristics of the participants are as laid out in Table 1. As expected, the cases had significantly higher systolic, diastolic, mean arterial and pulse pressure compared with the controls. A significantly lower baseline heart rate was observed in the cases compared to the controls (81.5 ± 15.4 vs 87.9 ± 10.8 bpm; p < 0.001). There were significantly more mothers with a previous history of pre-eclampsia among the cases compared to the controls (21.4 vs 4.5%; p < 0.001). There was no difference in the percentage of nulliparous women between the cases and controls (p < 0.05).

**Table 1:** General characteristics of the study population

*Characteristic*	*Controls (n = 110)*	*Cases (n = 105)*	*p-value*
Maternal age (years)	25.4 ± 0.5	27.0 ± 0.8	0.089
Gestational age (weeks)	32.3 ± 0.4	30.8 ± 0.4	0.017*
BMI (kg/m^2^)	30.7 ± 0.5	33.1 ± 0.8	0.010*
Systolic BP (mmHg)	112.3 ± 1.3	140 ± 1.8	0.0000*
Diastolic BP (mmHg)	64.2 ± 0.9	84.2 ± 1.6	0.00008
Mean arterial pressure (mmHg)	79.1 ± 1.2	102.8 ± 1.5	0.0000*
Pulse pressure (mmHg)	48.2 ±1.4	55.9 ± 1.4	0.0001*
Baseline heart rate (bpm)	87.9 ± 1.0	81.5 ±1.5	0.0004*
Parity	0.95 ± 0.1	1.42 ± 0.2	0.033*
History of previous pre-eclampsia, n (%)	5 (4.5)	22 (21.4)	0.0003*
Nulliparity, n (%)	37 (33.3)	42 (40.8)	0.3221
HIV+, n (%)	28 (25.2)	38 (36.9)	0.076
Family history of HPT, n (%)	31 (28)	37 (36)	0.251
Family history of DM, n (%)	25 (23)	15 (14.6)	0.162

BMI, body mass index; HPT, hypertension; DM, diabetes mellitus.

Women with pre-eclampsia were found to have significantly lower RHI [1.70 (1.04–3.61) vs 1.81 (1.18–4.62) au; p < 0.05] and log-transformed RHI [0.31 (–0.03–1.24) vs 0.48 (0.00–1.87) au; p < 0.01) compared to normotensive controls. Augmentation index at 75 bpm [12.42 (–35.79–81.76) vs 2.76 (–33.17–23.86)%; p < 0.0001] and BPWA [543.66 (23.44–1939.8) vs 450.56 (16.12–1359.4) au; p < 0.01] were found to be higher among women with pre-eclampsia compared to the normotensive controls, as shown in Table 2.

**Table 2:** Vascular reactivity characteristics of the two groups

*Characteristic*	*Controls (n = 110)*	*Cases (n = 105)*	*p-value*
Reactive hyperaemia index	1.81	1.70	0.0269
(RHI) (au)	(1.18–4.62)	(1.04–3.61)	
Log-transformed RHI	0.48	0.31	0.0034
(F-RHI)	(0.00–1.87)	(–0.03–1.24)	
Baseline pulse-wave	450.56	543.66	0.0021
amplitude (au)	(16.12–1359.4)	(23.44–1939.8)	
Augmentation index	2.76	12.42	0.0000
@75 (%)	(–33.17–23.86)	(–35.79–81.76)	

## Relationship between RHI, PAlx and BPWA with maternal risk factors

**Fig. 1.**
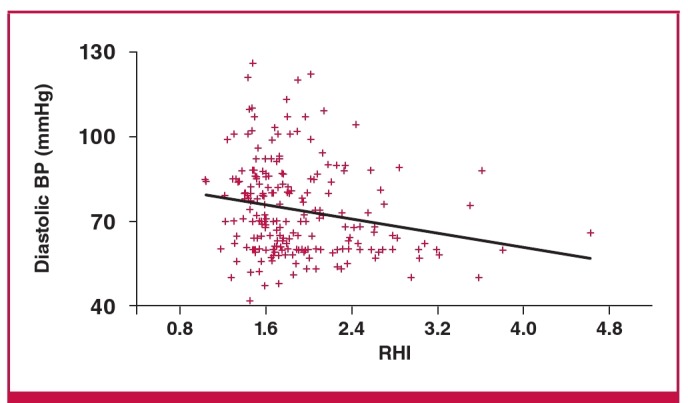
Relationship between diastolic blood pressure and RHI (p < 0.05).

**Fig. 2.**
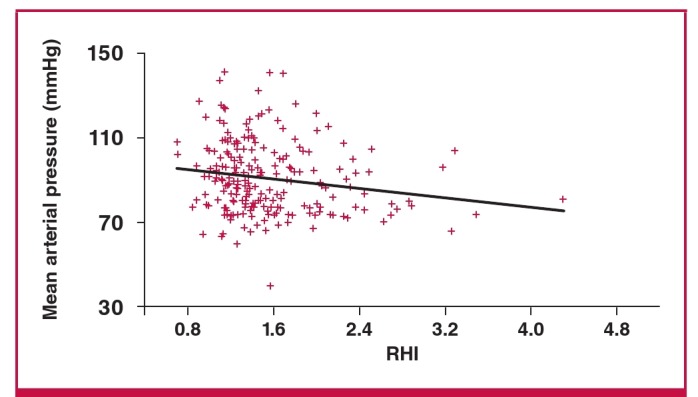
Relationship between mean arterial pressure and RHI (p = 0.0328).

**Fig. 3.**
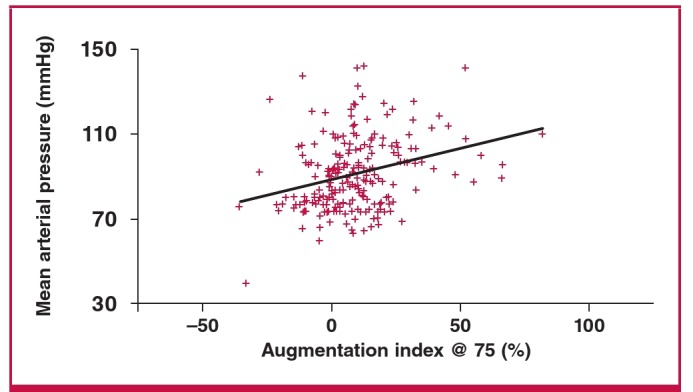
Relationship between mean arterial pressure and augmentation index @ 75 bpm (p = 0.0372).

On bivariate correlation analysis, there was a significant inverse relationship between RHI and diastolic blood pressure, parity and mean arterial pressure, and no relationship with maternal age, body mass index (BMI), systolic blood pressure, or total number of risk factors for pre-eclampsia, as seen in Table 3. On multiple regression analysis, there was a significant inverse correlation between diastolic blood pressure and RHI (r = –0.14, p < 0.05) and mean arterial blood pressure and RHI (coeff = –4.95053, SE = 2.29277; p < 0.05) as shown in Figs 1 and 2. The high diastolic blood pressure and mean arterial pressure were associated with lower RHI. Augmentation index was also positively associated with mean arterial pressure, as shown in Fig. 3.

**Table 3:** Relationship between RHI and maternal risk factors

*Factor*	*Coefficient (r)*	*p-value*
Maternal age	–0.095	0.171
Baseline heart rate	0.022	0.756
BMI	–0.122	0.078
Diastolic blood pressure	–0.210	0.0022
Parity	–0.138	0.045
Mean arterial pressure	–0.187	0.0066
Pulse pressure	0.051	0.461
Weight	–0.121	0.081
Systolic blood pressure	–0.124	0.072

**Fig. 4.**
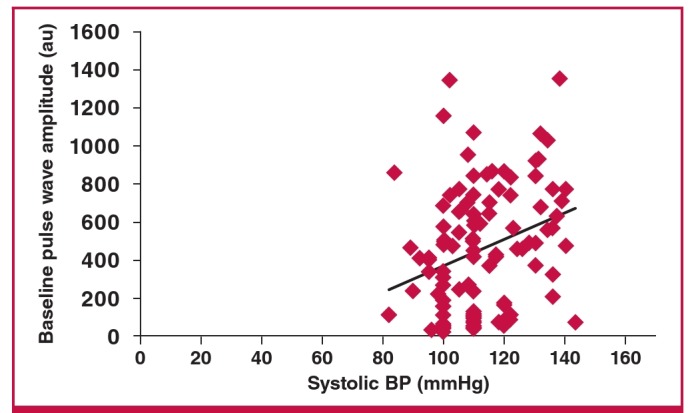
Relationship between systolic pressure and baseline pulse-wave amplitude.

BPWA was positively related to maternal age, BMI, parity, pulse pressure, weight and systolic blood pressure on univariate correlation, as seen in Table 4. On multiple regression analysis, systolic blood pressure was the only variable independently correlated with BPWA (r = 0.22, p = 0.0166), as seen in Fig. 4. Higher systolic blood pressure was therefore significantly associated with arterial stiffness.

**Table 4:** Relationship of BPWA and maternal risk factors

*Factor*	*Coefficient (r)*	*p-value*
Maternal age	0.141	0.0416
Baseline heart rate	–0.137	0.0475
BMI	0.238	0.0005
Diastolic blood pressure	0.079	0.2556
Parity	0.192	0.0052
Mean arterial pressure	0.123	0.076
Pulse pressure	0.169	0.014
Weight	0.209	0.0024
Systolic blood pressure	0.170	0.0136

## Differences in the study variables as per HIV status

The participants were divided into four groups, namely, (1) HIV-negative normotensive (A) (n = 83); (2) HIV-positive normotensive (B): (n = 27); (3) HIV-positive pre-eclamptic (C): (n = 38); and (4) HIV-negative pre-eclamptic (D) (n = 67) (Table 5). The Kruskal–Wallis test was used to analyse the differences between the four groups and Dunn’s multiple comparison posttest was used to check significance between the individual groups.

**Table 5:** Augmentation index @ 75 bpm between the four HIV groups.

**	*Normotensive*	*Normotensive*	*Pre-eclamptic*	*Pre-eclamptic*	**
*Characteristic*	*HIV + (n = 27)*	*HIV– (n = 83)*	*HIV+ (n = 38)*	*HIV– (n = 67)*	*p-value*
MAP	76.7	78.7	103.7	102.4	< 0.0001
(mmHg)	(64.0– 99.3)	(0– 103.3)	(64.3– 141.7)	(73.3– 142.0)	
RHI	1.79	1.84	1.67	1.70	0.1195
(au)	(1.18–3.21)	(1.22–4.62)	(1.22–3.61)	(1.04–2.84)	
BPWA	527.08 ± 68.272	417.69 ± 34.867	593.55 ± 64.295	601.78 ± 43.92	0.0072
(au)					
AIx @	8.02	0.82	15.09	10.53	< 0.0001
75 (%)	(–19.18–22.8)	(–50.7–23.6)	(–35.8–51.9)	(–27.9–81.6)	

MAP, mean arterial pressure; RHI, reactive hyperaemia index; BPWA, baseline pulse-wave amplitude; Aix, augmentation index @ 75 bpm.

For mean arterial pressure, significant differences were evident between HIV-positive normotensive and HIV-positive pre-eclamptic pregnant mothers (p < 0.001), HIV-positive normotensive and HIV-negative pre-eclamptic pregnant mothers (p < 0.001), HIV-negative normotensive and HIV-positive pre-eclamptic pregnant women (p < 0.0001), and between HIV-negative normotensive and the HIV-positive pre-eclamptic pregnant women (p < 0.001), respectively. Significant differences were observed for BPWA (p < 0.01) and augmentation index at 75 bpm (p < 0.0001) between the four groups.

**Fig. 5.**
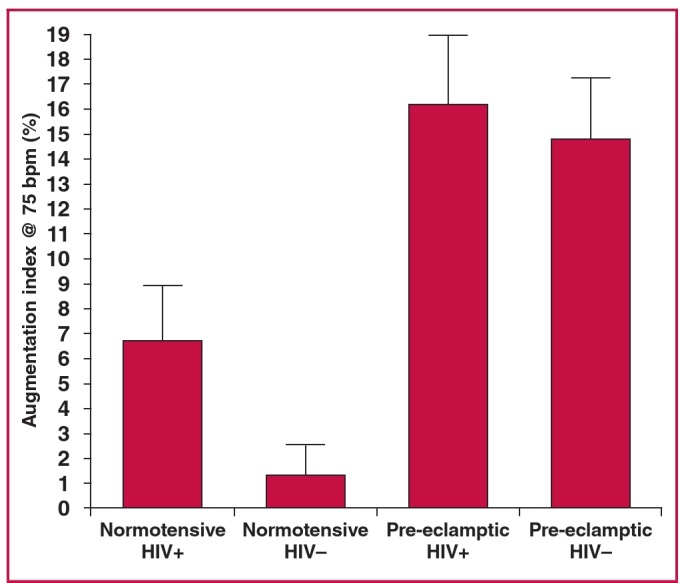
Augmentation index @ 75 bpm between the four HIV groups.

For BPWA, Dunn’s multiple comparison test revealed a significant difference only between HIV-negative normotensive and HIV-negative pre-eclamptic pregnant women (p < 0.01). For augmentation index, significant differences were observed between HIV-negative normotensive and HIV-positive pre-eclamptic pregnant women (p < 0.001) and between HIV-negative normotensive and HIV-negative pre-eclamptic pregnant women (p < 0.001), as shown in Fig. 5. RHI was lower in HIV-positive normotensive and HIV-positive pre-eclamptic women than in normotensive HIV-negative women, although this did not reach statistical significance (p = 0.1195).

## Discussion

In this study, we set out to assess whether PAT (through RHI) demonstrates endothelial dysfunction in pre-eclampsia. The RHI was found to be significantly lower in patients with pre-eclampsia compared to normotensive controls. Since RHI is endothelium dependent, these results indicate that there is indeed endothelial dysfunction in rural African women with pre-eclampsia, therefore confirming what has been reported in other populations. To our knowledge, this is the first report involving rural black African women.

Endothelial dysfunction is known to be the central mechanism in the pathophysiology of pre-eclampsia.^3^ Several reports have demonstrated that FMD is significantly reduced in patients with pre-eclampsia when compared with normotensive controls,^18^-^20^ confirming that pre-eclampsia is associated with endothelial dysfunction. Although FMD measurement is still regarded as the gold standard for assessing endothelial function in pregnancy, it has several limitations, including the need for an experienced sonographer, a good-quality ultrasound machine, and the need for intra-arterial injections. It is therefore not easy to adapt the method for use in assessing endothelial function in large numbers of patients in a clinic setting.

We have shown in this study that the EndoPAT 2000 can be used successfully to assess endothelial function in pregnant subjects by measuring the RHI. Although the EndoPAT 2000 itself is a fairly expensive machine, it is less invasive, much easier to use, does not require extensive training and it can be used to assess large numbers of patients rapidly and reliably, even in a clinic setting.

Although not many studies have tested endothelial function in pregnancy using EndoPAT 2000, our results are in agreement with the study done by Yinon et al. in 2006, who examined 17 women at the time of diagnosis of pre-eclampsia (mean gestation 32 weeks) and compared them with 25 women with normotensive pregnancies. They found that women with pre-eclampsia had significantly lower RHI values (1.5 ± 0.1 vs 1.8 ± 0.1) compared to uncomplicated pregnancies.

The results of this study with much larger number of subjects clearly indicate that RHI, as measured using the EndoPAT, can be used as an adjunct to blood pressure measurement in assessing endothelial dysfunction in pre-eclampsia. However, as endothelial dysfunction is known to precede clinical pre-eclampsia, the important question is, can the EndoPAT 2000 be used for screening and identifying patients before the onset of clinical pre-eclampsia? Carty^15^ followed up a cohort of patients in Scotland from the first trimester all through pregnancy to postpartum, but did not find any difference in RHI between women who went on to develop pre-eclampsia and normotensive pregnancies, both at 16 and 28 weeks’ gestation. This, however, does not rule out the possibility that RHI might still be useful in either early identification or prediction of pre-eclampsia in larger studies, as the search for predictors of pre-eclampsia continues in earnest.

If it were demonstrated that RHI, as measured by the EndoPAT 2000, can be used as a predictor of pre-eclampsia, then the test would become much more cost-effective and cheaper. A prospective cohort study of normotensive pregnant women recruited in the first or early second trimester and followed until delivery is planned to determine whether RHI measurement can be used as a predictor of pre-eclampsia.

When participants with early-onset pre-eclampsia were compared with those with late-onset pre-eclampsia, there was no statistically significant difference in RHI between patients with early- and late-onset pre-eclampsia (p > 0.05). Although the numbers are small, this finding suggests that endothelial dysfunction is indeed present in both early- and late-onset pre-eclampsia, and that this may be the central mechanism involved in the pathophysiology of both early- and late-onset pre-eclampsia.

Although some authors have suggested that early-onset pre-eclampsia is a result of impaired placentation,^21^ while lateonset pre-eclampsia is as a result of maternal predisposition,^22^ both are associated with endothelial dysfunction. As no one has as yet discovered the cause of pre-eclampsia, it is difficult to attach more significance to this attempt to separate early- from late-onset pre-eclampsia.

An inverse relationship observed between FMD and baseline vascular dimension has been reported from previous studies, where peak arterial dilation is a function of baseline vessel diameter and smaller vessels dilate more than larger ones.^23^ In our study, we found an inverse correlation between RHI and BPWA in the entire study population (normotensive and pre-eclamptics together), as well as within individual groups. Similar results were recently reported by Carty^15^ and Heffernan et al.^24^ in their studies on healthy volunteers as well as patients with coronary artery disease. The authors found that BPWA was positively related to baseline arterial diameter and in turn, inversely related to RHI. This means that smaller brachial arteries have low BPWA and subsequently a high RHI, and vice versa.

It has been suggested that in individuals with variable brachial geometry, adjusting RHI for brachial artery diameter may be needed to accurately examine microvascular endothelial function using PAT.^24^ Although baseline artery diameters were not measured in our study, several studies have shown no difference in baseline brachial artery diameters between normotensive pregnancy and pre-eclampsia.^25^-^27^ In view of these results from other studies, it can be hypothesised that the inverse relationship between RHI and BPWA observed in this study could be due to factors other than the brachial artery diameter. Further studies will need to be done to determine the factors responsible for the inverse relationship between RHI and baseline brachial artery diameter in pregnancy.

An inverse relationship was found between RHI, mean arterial pressure and diastolic blood pressure in the entire study population. No relationship was observed between RHI and systolic blood pressure. Other studies have reported conflicting relationships between RHI and mean arterial pressure, diastolic and systolic blood pressure. Truschel et al.^28^ showed a positive correlation (among men and non-pregnant women) between RHI and systolic blood pressure, whereas Konttinen et al.^29^ and Hamburg et al.11 showed no correlation between diastolic, systolic and mean arterial pressure and RHI. BPWA was, however, found to be positively correlated with systolic blood pressure.

Hamburg et al. suggested that systolic blood pressure may have a limited effect on the distal microcirculation, whereas it had a predominant effect on BPWA without additional modification of the hyperaemic response, when presented as a ratio.^11^ Since RHI is negatively correlated with BPWA (which in turn is positively correlated with systolic blood pressure), by extrapolation, RHI would be negatively correlated with systolic blood pressure. While assessing racial differences in microcirculatory function, Morris et al. also found mean arterial pressure to be negatively associated and an independent predictor of RHI.^30^

In pregnancy, a similar correlation between RHI and mean arterial pressure was found in a study done in Israel assessing the relationship of pre-eclampsia with sleep-disordered breathing.^17^ This relationship illustrates the impact of blood pressure on microvascular function. Pre-eclampsia is characterised by generalised vasoconstriction, an increase in peripheral resistance, platelet activation, reduced plasma volume and organ hypoperfusion.^31^,^32^ The aetiology is still unclear, although evidence suggests that increments in blood pressure may reflect endothelial dysfunction, with the inability of the endothelial cells to release relaxing factors that cause vasodilatation.^33^,^34^ Endothelial dysfunction is known to lead to the widespread clinical features of pre-eclampsia. Results from our study therefore are in agreement with previous studies that found endothelial dysfunction in women with pre-eclampsia.^35^,^36^

Traditionally, PAlx is obtained from pressure waveforms via applanation tonometry of the carotid or radial arteries. Recently, it has been demonstrated that PAlx measured from digital pulse-wave volumes by peripheral PAT correlated with that from applanation tonometry^14^,^37^ in diabetes and idiopathic scleroderma associated with pulmonary arterial hypertension. Carty^38^ and Namugowa and Meeme^39^ also reported that EndoPAT augmentation index correlated with radial artery applanation augmentation index (measured using the SphygmoCor) in pregnancy. However, they cannot be used interchangeably since the actual values do not match, as they are obtained from two distinct vascular beds via two different methods.

Patvardhan et al.^40^ found that augmentation index derived from PAT correlated with cardiovascular risk factors. In this study, we found that heart rate-corrected augmentation index (Alx@75) was higher in pre-eclampsia compared to that in normotensive pregnancy, indicating arterial stiffness. Similar results have been reported by others.^41^-^45^

Normal pregnancy is a profoundly vasodilated state.^46^ This vasodilated state could be due to the remarkable maternal cardiovascular adaptation to pregnancy, which is the attenuated systemic pressor response to vasoconstrictors, including angiotensin II and norepinephrine.^47^,^48^ A reduction in vascular compliance in pre-eclampsia is an indication of vascular stiffness, as is seen in non-pregnant patients with chronic hypertension, vascular disease or diabetes.^49^-^52^ It may also indicate vasoconstriction, most likely due to endothelial dysfunction, since systemic response to vasoconstrictors such as angiotensin II and norepinephrine is not attenuated but augmented in pre-eclampsia.^48^

The reactive hyperaemia index, as a measure of endothelial dysfunction, was lowest in HIV-positive pre-eclamptics (1.67) and highest in HIV-negative normotensive controls (1.84). In HIV-positive normotensive controls, the RHI was 1.79 and it was 1.70 in HIV-negative pre-eclamptic cases. Although these differences did not achieve statistical significance, a trend towards a lower reactive hyperaemia index can be seen in the HIV-positive patients, indicating increasing levels of endothelial dysfunction. Arterial stiffness, as measured by the augmentation index was significantly worse (p < 0.000) in the HIV-positive pre-eclamptic and normotensive women than in the HIV-negative pre-eclamptic and normotensive women. Arterial stiffness is also a measure of endothelial dysfunction, therefore indicating that HIV infection is indeed associated with endothelial dysfunction in both normotensive and pre-eclamptic patients.

Considering that vasodilation, upon which the EndoPAT depends, is influenced by temperature, efforts were made to conduct the measurements in temperature-controlled rooms for all participants. Due to insufficient funds, we were not able to compare EndoPAT results with a blood marker of endothelial dysfunction, but such a study is planned.

## Conclusion

This study has shown that there is impaired endothelial function in rural African women with pre-eclampsia, based on the low RHI and increased arterial stiffness, as measured by the BPWA and augmentation index, compared to normotensive pregnancies. This clearly indicates that RHI, BPWA and Alx@75, as measured using the EndoPAT, can be used for assessing endothelial dysfunction in pre-eclampsia. Although PAT could be used as an adjunct to blood pressure measurement in assessing patients with pre-eclampsia, the EndoPAT is a relatively expensive piece of equipment.

The EndoPAT 2000 may earn its worth if it could detect endothelial dysfunction and hence be used for screening patients before the onset of hypertension and proteinuria. This will need to be assessed in a prospective, cohort study of pregnant women from the first or early second trimester until delivery, in order to determine whether a reduced RHI can be detected before the onset of pre-eclampsia. Such a study is planned.
